# ^18^F‐fluoromisonidazole uptake in advanced stage non‐small cell lung cancer: A voxel‐by‐voxel PET kinetics study

**DOI:** 10.1002/mp.12416

**Published:** 2017-07-21

**Authors:** Daniel R. McGowan, Ruth E. Macpherson, Sara L. Hackett, Dan Liu, Fergus V. Gleeson, W. Gillies McKenna, Geoff S. Higgins, John D. Fenwick

**Affiliations:** ^1^ Cancer Research UK/MRC Oxford Institute for Radiation Oncology Gray Laboratories Department of Oncology University of Oxford Oxford UK; ^2^ Radiation Physics and Protection Oxford University Hospitals NHS Foundation Trust Oxford UK; ^3^ Department of Radiology Oxford University Hospitals NHS Foundation Trust Oxford UK; ^4^ Department of Oncology Oxford University Hospitals NHS Foundation Trust Oxford UK

**Keywords:** compartment modeling, dynamic PET, FMISO, kinetics analysis, NSCLC

## Abstract

**Purpose:**

The aim of this study was to determine the relative abilities of compartment models to describe time‐courses of ^18^F‐fluoromisonidazole (FMISO) uptake in tumor voxels of patients with non‐small cell lung cancer (NSCLC) imaged using dynamic positron emission tomography. Also to use fits of the best‐performing model to investigate changes in fitted rate‐constants with distance from the tumor edge.

**Methods:**

Reversible and irreversible two‐ and three‐tissue compartment models were fitted to 24 662 individual voxel time activity curves (TACs) obtained from tumors in nine patients, each imaged twice. Descriptions of the TACs provided by the models were compared using the Akaike and Bayesian information criteria (AIC and BIC).

Two different models (two‐ and three‐tissue) were fitted to 30 measured voxel TACs to provide ground‐truth TACs for a statistical simulation study. Appropriately scaled noise was added to each of the resulting ground‐truth TACs, generating 1000 simulated noisy TACs for each ground‐truth TAC. The simulation study was carried out to provide estimates of the accuracy and precision with which parameter values are determined, the estimates being obtained for both assumptions about the ground‐truth kinetics.

A BIC clustering technique was used to group the fitted rate‐constants, taking into consideration the underlying uncertainties on the fitted rate‐constants. Voxels were also categorized according to their distance from the tumor edge.

**Results:**

For uptake time‐courses of individual voxels an irreversible two‐tissue compartment model was found to be most precise. The simulation study indicated that this model had a one standard deviation precision of 39% for tumor fractional blood volumes and 37% for the FMISO binding rate‐constant.

Weighted means of fitted FMISO binding rate‐constants of voxels in all tumors rose significantly with increasing distance from the tumor edge, whereas fitted fractional blood volumes fell significantly. When grouped using the BIC clustering, many centrally located voxels had high‐fitted FMISO binding rate‐constants and low rate‐constants for tracer flow between the vasculature and tumor, both indicative of hypoxia. Nevertheless, many of these voxels had tumor‐to‐blood (*TBR*) values lower than the 1.4 level commonly expected for hypoxic tissues, possibly due to the low rate‐constants for tracer flow between the vasculature and tumor cells in these voxels.

**Conclusions:**

Time‐courses of FMISO uptake in NSCLC tumor voxels are best analyzed using an irreversible two‐tissue compartment model, fits of which provide more precise parameter values than those of a three‐tissue model. Changes in fitted model parameter values indicate that levels of hypoxia rise with increasing distance from tumor edges.

The average FMISO binding rate‐constant is higher for voxels in tumor centers than in the next tumor layer out, but the average value of the more simplistic *TBR* metric is lower in tumor centers. For both metrics, higher values might be considered indicative of hypoxia, and the mismatch in this case is likely to be due to poor perfusion at the tumor center. Kinetics analysis of dynamic PET images may therefore provide more accurate measures of the hypoxic status of such regions than the simpler *TBR* metric, a hypothesis we are presently exploring in a study of tumor imaging versus histopathology.

## 1 Introduction

The radiotracer ^18^F‐fluoromisonidazole (FMISO) diffuses passively into cells, where it is reduced and in hypoxic environments is irreversibly bound, allowing hypoxic tumor subvolumes to be imaged via positron emission tomography (PET) of FMISO uptake.^1^, ^2^, ^3^ Survival rates for patients with locally advanced non‐small cell lung cancer (NSCLC) are currently poor following chemoradiotherapy (CRT) and might be improved by selectively boosting radiation doses delivered to these hypoxic subvolumes.^3^, ^4^, ^5^, ^6^ To do this, most effectively requires knowledge of the degree of hypoxia, which can be estimated either from uptake levels in single FMISO images collected 2–4 h after tracer injection,^7^ or by analyzing the kinetics of FMISO uptake in dynamic sequences of PET images (dPET) in order to determine rate‐constants of FMISO intracellular binding. Generally for static imaging, a tumor‐to‐blood ratio (*TBR*) threshold of 1.4 is used to indicate hypoxia.^7^ FMISO kinetics analysis can be performed at the whole tumor level or voxel‐by‐voxel, and for head‐and‐neck cancers has generated indices that correlate with RT outcomes.^8^

**Figure 1:**
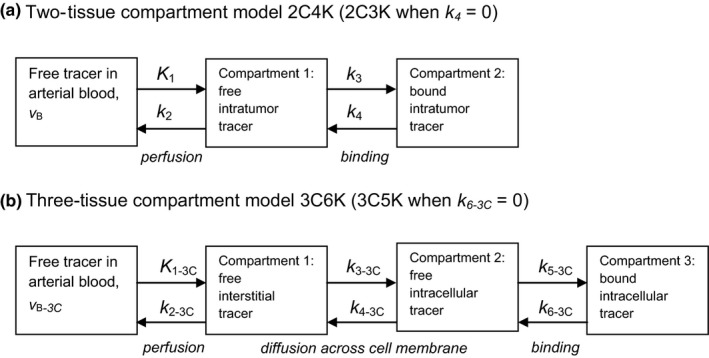
Two‐ (a) and three‐ (b) tissue compartment models. Flow rates from one compartment to another are defined by rate‐constants (*k* values), tracer concentrations in the various compartments, and compartment volumes.^12^ The fraction of the tumor volume occupied by blood is denoted in each model as *ν*_*B*_.

Several methods have been used to analyze dPET data, the most common approaches being compartment modeling^9^ and the spectral analysis technique developed by Cunningham and Jones.^10^ In compartment modeling, time‐courses of tumor tracer uptake are often described using a model comprising two‐tissue compartments representing intra‐tumor free and bound tracer, which, together with blood‐borne tracer, account for the total tumor tracer uptake.^11^, ^12^ Figure 1 schematically illustrates this model alongside an alternative with three‐tissue compartments, the additional compartment describing the tumor interstitium lying between the vasculature and cells.^13^ Tracers generally diffuse from blood vessels into the interstitium and are then transported across the cell membrane to be bound intracellularly. Each of these processes potentially has different rate‐constants. This means that modeling time‐courses of tumor tracer uptake sequentially (as in the three‐tissue compartment model) may differ to that by merging the processes (two‐tissue compartment model) and so both are investigated in this work.

We denote by *x*C*y*K, a model comprising a linear chain of *x*‐tissue compartments (excluding blood‐borne tracer) and *y* rate‐constants: thus two‐ and three‐compartment models with reversible flow between each compartment are named 2C4K and 3C6K. In order to associate rate‐constants with particular models we add the subscript *x*C to their names, except for rate‐constants of the two‐tissue compartment model. Binding of FMISO is generally considered irreversible, and therefore alongside the 2C4K and 3C6K models, it is also useful to explore simpler two‐ and three‐compartment models 2C3K and 3C5K in which the rate‐constant of flow from bound to unbound tracer compartments is set to zero. In common with other work, the first rate‐constant (*K*_1_) is capitalized indicating it has units of ‘mL min^−1^ mL^−1^’ tissue compared to ‘min^−1^’ for the other rate‐constants. Compartment configurations other than linear chains, such as branching models, are also possible, however, transformation analysis can sometimes reduce these models to equivalent models with linear chains of compartments.^14^

In this work, we investigate which linear chain compartment model best describes time‐courses of FMISO uptake in individual voxels of tumors in patients with non‐small cell lung cancer (NSCLC) imaged using dynamic PET. We use statistical simulations to identify the model whose fits to the time‐course data provide the most precise estimates of tracer kinetics rate‐constants. We hypothesize that perfusion and potentially hypoxia may change with distance from the tumor edge and so we explore the relationship between fitted rate‐constant values and voxel distance from the tumor edge, an issue that has not been studied to date.

While dynamic FMISO PET scans have previously been analyzed using compartment modeling,^8^, ^15^, ^16^, ^17^ such work has not been carried out for NSCLC patients. With the exception of Casciari et al. who proposed a detailed model based on the chemical pathways of FMISO reduction and binding, researchers have not explored models more complex than 2C4K in connection with FMISO uptake.^2^ The 2C3K model (or close variants) has mostly been used, with one group using the reversible 2C4K model to investigate the kinetics of FMISO uptake by brain tissue.^18^

## 2 Materials and methods

### 2.A. FMISO‐PET image data

Pre‐clinical research has shown that the investigational drug Buparlisib (Novartis, Basel, Switzerland) reduces tumor hypoxia *in vivo*.^19^ A clinical trial underway in Oxford, NCT02128724, has the primary aim of determining the maximum tolerated dose of Buparlisib in NSCLC patients treated palliatively using radiotherapy, and the secondary goal of validating the pre‐clinical results in these patients, who are imaged using FMISO‐PET at baseline and 7 days after administration of the drug without any other intervention. Following the second FMISO‐PET scan, the patients commence palliative thoracic radiotherapy consisting of 20 Gy delivered in five fractions over a week. The study has been approved by the local ethics committee and signed informed consent obtained from all patients.

Patients were imaged supine with their arms by their side using a Discovery 690 PET/CT scanner (GE Healthcare, Milwaukee, USA). They were injected with 370 MBq FMISO 30 s into PET imaging, which continued for 45 min and resumed for 10 min intervals at 2 and 4 h post‐injection. Prior to each PET acquisition a CT scan was performed for localization and PET attenuation correction. The PET data were reconstructed using a time‐of‐flight ordered subset expectation maximization algorithm (VPFX, GE Healthcare) with a standard 6.4 mm Gaussian filter applied post‐reconstruction (with resulting image resolution approximately 7 mm). Respiratory motion correction was not performed, see details in the Discussion. The first 45 min of data were binned into two parallel time sequences, S1 (1 × 30 s, 12 × 5 s, 6 × 10 s, 6 × 30 s, 10 × 60 s, 6 × 300 s) and S2 (1 × 30 s, 60 × 1 s, 12 × 10 s, 4 × 30, 10 × 60 s, 6 × 300 s), and reconstructed as images on a matrix of 5.5 × 5.5 × 3.3 mm^3^ voxels. Data collected during the two later 10‐min intervals were processed as single frames. The PET/CT images collected at 2 and 4 h post‐injection were rigidly registered (CT‐to‐CT) to the dynamic PET/CT image using an automatic registration tool in the Hermes Hybrid Viewer (Hermes Medical Solutions AB, Stockholm, Sweden), followed by manual adjustment (matching to the tumor region) when required.

Primary tumors were outlined on the images by an experienced radiologist. Cylindrical blood volumes of diameter 10 mm were defined within the central part of the descending aorta, whose typical diameter is 25 mm, on five or more consecutive PET axial slices.^20^ TACs representing time‐courses of mean tracer activity concentrations within each of the tumor volumes‐of‐interest (VOIs) and blood volumes were obtained from PET sequences S1 and S2 respectively. Activity data from the 10‐min frames collected at 2 and 4 h post‐injection were appended to the TACs.

**Figure 2:**
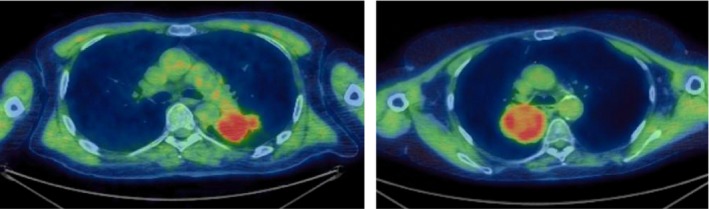
Two example FMISO PET/CT images 4 h p.i. (pre‐Buparlisib) on an SUV scale 0–3 for two different patients. [Color figure can be viewed at wileyonlinelibrary.com]

Kinetic analysis was performed on all 24 662 individual voxel FMISO time‐activity curves (TACs) obtained from nine primary tumors in nine patients, each imaged twice (Table 1). The primary tumors are in a variety of anatomical locations within the lung, generally in the upper lobe. Patient 2 had a necrotic center. Example images of FMISO uptake at 4 h post‐injection (p.i.) are shown in Fig. 2. Differences between tracer uptake before and after drug administration are not reported here, as this information will be published on completion of the trial.

**Table 1:** Details of the imaged patients and primary tumors analyzed. ‘PS’ denotes performance status, ‘adeno’ adenocarcinoma, and ‘squam’ squamous cell

Patient	Sex	PS	Stage	Histological type	Primary tumor volume (mL)
1	F	1	IV	Adeno	13
2	M	1	IIIa	Squam	510
3	M	1	IV	Adeno	105
4	M	1	IV	Squam	29
5	F	1	IV	Adeno	135
6	F	1	IV	Squam	180
7	F	1	IV	Adeno	44
8	M	0	IV	Squam	188
9	M	1	IV	Squam	40

### 2.B. Kinetics analysis and model fitting

Kinetics analysis was carried out using PMOD software (PMOD Technologies, Zürich, Switzerland). Input functions describing tracer flow into tumors were obtained by fitting a standard model of tracer plasma concentration developed by Feng et al.^21^, ^22^ to decay‐corrected blood TACs, using the Marquard–Levenberg algorithm to achieve the best match between the measured and modeled blood data as gauged by the weighted sum‐of‐squares:

(1) $$SS=\sum_{i=1}^{N}w_{i}{\left(C_{PET}\left(t_{i}\right)-C_{model}\left(t_{i}\right)\right)}^{2}$$

in which *C*_*PET*_(*t*_*i*_) and *C*_*model*_(*t*_*i*_) are the imaged and modeled mean activity concentrations at time *t*_*i*_, the mid‐point of the *i*th of *N* time‐points, and *w*_*i*_ is the relative weighting factor for the time‐point, given by

(2) $$w_{i}=\Delta t_{i}\;\exp\left(-\lambda t_{i}\right)/C_{PET}\left(t_{i}\right)$$

where Δ*t*_*i*_ is the duration of the *i*th frame and *λ* is the decay constant for ^18^F.^13^, ^22^

Mathematical representations of the two‐ and three‐tissue compartment models of tumor tracer uptake were then fitted to the decay‐corrected FMISO uptake TACs measured for single voxels, given the tracer influxes described by the input functions.^12^, ^13^ Fitted model parameters were optimized to minimize the weighted square difference defined by Eq. (1), now between imaged and modeled tumor TACs. Model fitting was initiated from 100 randomly generated sets of starting values to attempt to reach global rather than local best fits.

A parameter termed *k*_flux_ was calculated:

(3) $$k_{flux}=\frac{K_{1}k_{3}}{k_{2}+k_{3}}$$

(4) $$k_{flux-3C}=\frac{K_{1}k_{3}k_{5}}{k_{2}k_{4}+k_{2}k_{5}+k_{3}k_{5}}$$

for two‐ (*k*_flux_) and three‐tissue (*k*_flux−3C_) compartment models respectively. For irreversible models (*k*_4_ = 0 or *k*_6−3C_ = 0) *k*_*flux*_ represents the rate of tracer uptake given a steady‐state unit input, or equivalently the tumor uptake at an infinitely late time‐point divided by the area under the real input‐function.^13^

### 2.C. Assessment of model fits

The Wald‐Wolfowitz runs test was used to determine the adequacy of descriptions of FMISO uptake TACs provided by compartment model fits.^23^, ^24^ To further assess the relative abilities of the different models to describe the data, we used the Akaike information criterion (AIC)^25^ corrected for small sample size^26^ and the Bayesian information criterion (BIC).^26^ Information criteria characterize the relative abilities of different models to describe data, penalizing more highly parameterized models which are likely to over fit data, in favor of models that are more parsimonious but retain sufficient parameters to adequately describe the data.

AIC and BIC values were calculated by adding to a scaled form (*SS*_*scale*_) of the sum‐of‐squares of Eq. (1) the terms 2*M *+ 2(*M *+ 1)(*M *+ 2)/(*N *− *M *− 2) and *M* ln*N* respectively, where *M* denotes the number of model parameters in a model and *N* the number of data‐points fitted. The scaled sum‐of‐squares used is given by

(5) $$SS_{scale}=\frac{SS}{SF}=\sum_{i=1}^{N}w_{i}\frac{{\left(C_{PET}\left(t_{i}\right)-C_{model}\left(t_{i}\right)\right)}^{2}}{SF}=\sum_{i=1}^{N}\frac{{\left(C_{PET}\left(t_{i}\right)-C_{model}\left(t_{i}\right)\right)}^{2}}{\sigma_{est\;C_{PET}\left(t_{i}\right)}^{2}}$$

where *SF* is a scale factor and $\sigma_{est\;C_{PET}\left(t_{i}\right)}^{2}$ an estimate of the statistical variance of the imaged mean activity concentration of the VOI, given by

(6) $$\sigma_{est\;C_{PET}\left(t_{i}\right)}^{2}=\left(SF\;\;C_{PET}\left(t_{i}\right)\exp\left(\lambda t_{i}\right)\right)/\Delta t_{i}$$

with the best model having the lowest calculated AIC or BIC (scaled) score.

The number of radioisotope decays occurring within a VOI is Poisson‐distributed, and if all decays were detected during PET imaging and accurately attributed to the VOI, *SF* would be the reciprocal of the VOI volume. In practice, however, *SF* takes a larger value due to noise‐propagation during image reconstruction and because many decays go undetected.^13^, ^22^, ^24^ For good fits to TAC data and accurate estimates of the variance of imaged activity concentrations, *SS*_*scale*_ has a chi‐square distribution with (*N*‐*M*) degrees‐of‐freedom.

For three randomly selected voxels within each tumor volume, we therefore estimated *SF* as the value of *SS*/(*N*‐*M*) obtained from the fit of the model having the lowest number of parameters of any model passing the runs test for that TAC. For each tumor volume, the average *SF* value obtained for the three selected voxels was then used for all the voxels within the volume and all model fits, as it was impractical to calculate an individual *SF* for every voxel. The average voxel SF with the weighting factor used here was 9.2 ± 4.1 (one standard deviation, s.d.). Similarly, rather than calculating AIC and BIC scores for all 24 662 voxels, we instead calculated them for a 30 voxel subset comprising the three voxels randomly selected from each of 10 tumor volumes.

It is also possible to use unscaled (logarithmic) forms of the AIC and BIC;^13^ we have calculated values for these quantities too, denoting them AIC and BIC (unscaled).

### 2.D. Accuracy and precision of fitted rate‐constants

The compartment model providing the best description of tumor TACs may not be the one whose fitted parameter values lie closest to the true tracer uptake rate‐constants, the determination of which is the primary goal of kinetics analysis. Therefore, we have used a statistical simulation procedure to assess which model produces the most accurate and precise rate‐constant estimates.

For the voxel‐by‐voxel analysis, 2C3K and 3C5K model fits to the 30 voxel TACs used in the AIC/BIC analysis were taken as ground‐truth, with the corresponding parameter values of these fits taken as ground‐truth rate‐constants (see Table III).

**Figure 3:**
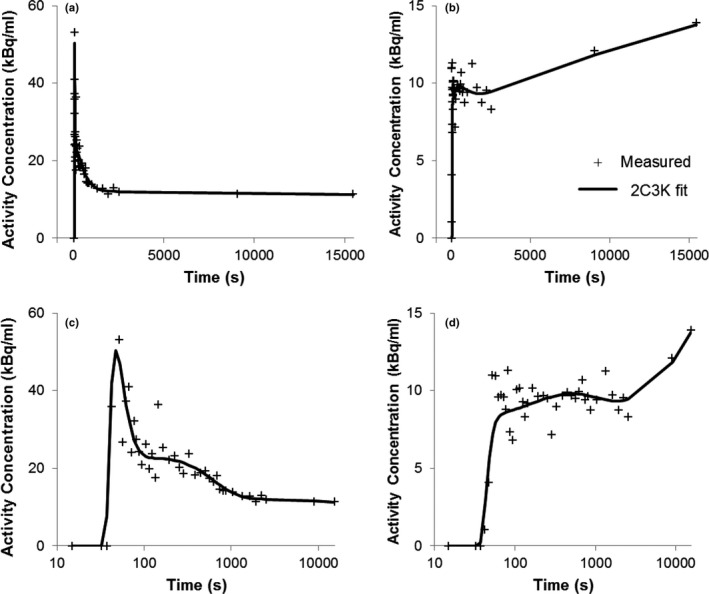
Fits of the 2C3K model to two example voxel TACs. Time post‐injection is plotted on linear (a/b) and logarithmic (c/d) scales.

For each of the resulting 60 ground‐truth TACs, 1000 noisy TACs were simulated by adding normally distributed random variables, with variances given by Eq. (6), to the activity concentrations of the individual time‐frames.^13^, ^27^, ^28^, ^29^ These simulated TACs had similar noise levels to the measured TACs (example measured TACs shown in Fig. 3). The simulated TACs were then fitted using the 2C3K and 3C5K models.

The simulated noise introduces random uncertainties and systematic error (bias) into fitted parameter values, adding to any underlying bias that results from mismatches between the fitted models and the ground‐truth TACs used to generate the simulated TACs. For each ground‐truth TAC, the bias in a fitted model parameter was calculated as the difference between the mean parameter value in the fits to the 1000 simulated noisy TACs and the ground‐truth parameter value, while the variance was obtained from the spread around the mean parameter value.

For both ground‐truth models, individual biases obtained for the 30 associated ground‐truth TACs were combined to determine the mean bias (MB) and variance of bias values ($\sigma_{B}^{2}$). The mean variance ($\sigma_{P}^{2}$) was calculated for each parameter as the average of the parameter variances obtained for the 30 ground‐truth TACs. For any particular measured TAC, the difference between the mean bias and the unknown individual bias in the fit to that TAC combines with the statistical uncertainty to create a total 1 standard deviation uncertainty on a fitted parameter given by

(7) $$\sigma_{T}={\left(\sigma_{B}^{2}+\sigma_{P}^{2}\right)}^{1/2}$$

This uncertainty cannot be eliminated and is therefore of particular concern, whereas *MB* represents a constant offset on all fitted values which would cancel if parameter values were interpreted in the light of studies of associations between outcomes or biomarkers and previous parametric images with the same mean bias.^3^, ^8^ Alternatively for some imaging investigations carried out pre‐ and post‐intervention, the bias error on individual voxels might perhaps be constant, in which case σ_P_ would be the uncertainty of concern.

We calculated *MB*, σ_*B*_, σ_*P*_, and σ_*T*_ values for the model parameter *ν*_*B*_ which describes the fractional tumor volume occupied by the blood, for the *k*_*flux*_ and *k*_*flux*−3C_ composite flux‐constants of the fitted two‐ and three‐tissue compartment models,^13^, ^22^ and for all individual rate‐constants of the fitted models for which directly related rate‐constants exist within the ground‐truth compartment models. Some rate‐constants of fitted models are not directly related to any single ground‐truth model parameter: for example, processes described by the two‐tissue compartment *K*_1_ parameter are split between rate‐constants *K*_1−3C_ and *k*_3−3C_ in three‐tissue compartment models. For such rate‐constants, we have calculated σ_*P*_ values alone.

### 2.E. Clustering of fitted rate‐constant data

When useful, fitted parameter values for any single rate‐constant parameter obtained for all 24 662 tumor voxels were clustered using in‐house MATLAB code (version R2014a, MathWorks, Natick, MA, USA) according to an information criterion‐based method of Liu et al.^16^ extended to factor patient‐to‐patient bias variation into estimates of total uncertainties on fitted parameter values.

This clustering consisted of the following steps:

1. A number of discrete cluster levels *K* was assumed.
2. Parameter values *x*_*c*_ of each voxel *c* (1, …, *N*_*T*_) were initially grouped into *K* discrete levels of value *X*_*cluster−b*_ (*b *= 1, …, *K*) using a weighted *k*‐means clustering algorithm. The weights associated with each point, *w*_c_, were defined as
  (8) $$w_{c}={\left({\sigma_{B}}^{2}+{\sigma_{c}}^{2}\right)}^{-1}$$

where σ_B_ is the variability (one s.d.) of patient‐specific bias for that parameter (from Table IV) and σ_c_ is the statistical uncertainty (one s.d.) on the fitted parameter value (*x*_*c*_).

1. Matrix *Z*_*bc*_ was generated by assigning the matrix element (*b*,*c*) a value of 1 if voxel *c* was included in cluster level *b*, and 0 otherwise.
2. An iterative expectation maximization (EM) algorithm was used to refine the initial clustering, adjusting *X*_*cluster−b*_ and *Z*_*bc*_ values to maximize the mixture‐likelihood.
3. After convergence, *BIC* was calculated as BIC(*K*) = −2 ln *L*(*K*) + *K* ln*N*_*T*_. where *N*_T_ is the total number of voxels clustered.
4. Steps 1 to 5 were repeated for a range of *K* levels (typically 1–20).
5. The cluster grouping with the lowest BIC was taken to be the best clustered representation of the data after taking into account the underlying uncertainties on the fitted rate‐constants.

### 2.F. Distance categorization of fitted rate‐constants

In‐house MATLAB code (version R2014a, MathWorks, Natick, MA, USA) was used to determine the distance of each tumor voxel (dimension 5.5 × 5.5 × 3.3 mm^3^) from the nearest edge of the outlined tumor. Voxels were then separated into four distance categories: *edge* (the outermost shell of voxels), *outer* (voxel center up to 5.5 mm inside the tumor outline), *inner* (between 5.5 and 11 mm inside the outline), and *central* (greater than 11 mm inside the outline).

To assess whether average fitted parameter values varied with distance from the tumor edge, weighted means ($\overset{¯}{x}$) of the unclustered values were calculated for each distance category as

(9) $$\overset{¯}{x}=\frac{\sum_{d=1}^{N_{D}}w_{d}x_{d}}{\sum_{d=1}^{N_{D}}w_{d}}$$

where *x*_d_ is the parameter value for the *d*th of *N*_*D*_ voxels within a distance category and *w*_d_ the weighting as defined in Eq. (2). Statistical significances of differences between weighted means were assessed using Welch's *t*‐test for samples of unequal variance.

## 3 Results

### 3.A. Voxel‐by‐voxel kinetics analysis

Totaled AIC and BIC scores for fits to the TACs of the 30 voxel subgroup studied in the assessment of model performance are shown in Table 2, together with numbers of runs test passes. Fits of the 3C5K model passed the runs test for all these voxels, had lower total AIC and BIC scores than fits of the 2C3K, 2C4K, and 3C6K models, and had the lowest individual scores for more voxels than the other models.

**Table 2:** Summary of runs test results and AIC and BIC scores for model fits to 30 randomly selected voxel TACs. Lowest AIC and BIC scores are underlined, indicating the best model according to that measure. AIC and BIC values have been calculated using both the scaled and unscaled versions as indicated in the table

Model	2C3K	2C4K	3C5K	3C6K
Runs test passes from fits to all 30 TACs				
Runs passes	22	27	30	30
Information criteria summed for all statistical simulation TACs				
AIC (scaled)	1793	1500	1424	1508
BIC (scaled)	2002	1738	1687	1790
AIC (unscaled)	2826	2595	2473	2549
BIC (unscaled)	2999	2801	2702	2801
Numbers of TACs for which each model has the lowest scores				
AIC (scaled)	4	8	18	0
BIC (scaled)	8	7	15	0
AIC (unscaled)	4	6	20	0
BIC (unscaled)	8	7	15	0

Table 3 shows the mean rate‐constants used for the statistical simulation work. Table 4 shows statistical simulation results for ground‐truths represented by fits of the 2C3K and 3C5K models to measured TACs. When 2C3K model fits were used to represent the ground‐truth, 2C3K fits to the simulated data had lower mean biases and total uncertainties than 3C5K fits (e.g., 38% σ_T_ for *k*_3_ compared to 597% σ_T_ for *k*_5−3C_). Similarly, when the ground‐truth was instead represented by 3C5K model fits, 2C3K fits to the simulated data still generally had lower variances than 3C5K fits (although their mean biases were higher) and total uncertainties on 2C3K fit parameters were also generally lower or no worse than those on fitted 3C5K parameter values (e.g., 65% σ_T_ for *k*_3_ compared to 153% σ_T_ for *k*_5−3C_). Since mean bias can in principle be eliminated by appropriate normalization using previous imaging data, whereas variance and total uncertainty cannot, the 2C3K model appears the best option for fitting to single voxel TACs, providing the most precise fitted parameter estimates.

**Table 3:** Mean and standard deviations of the parameter values fitted to the 30 voxel TACs used as ground‐truth in the statistical simulations for both 2C3K and 3C5K models

2C3K	*v*_B_ (%)	*K*_1_ (mL min^−1^g^−1^)	*k*_2_ (min^−1^)			*k*_3_ (min^−1^)	*k*_*flux*_ (mL min^−1^g^−1^)
Mean	4.2	0.22	0.27			0.0016	0.0013
SD	5.6	0.098	0.11			0.0012	0.00096
3C5K	*v*_B‐3C_ (%)	*K*_1−3C_ (mL min^−1^g^−1^)	*k*_2−3C_ (min^−1^)	*k*_3−3C_ (min^−1^)	*k*_4−3C_ (min^−1^)	*k*_5−3C_ (min^−1^)	*k*_*flux*−3C_ (mL min^−1^g^−1^)
Mean	2.6	0.38	0.91	0.48	0.20	0.0027	0.00088
SD	4.3	0.29	0.76	0.50	0.13	0.0021	0.000015

**Table 4:** Estimates of accuracy and precision for parameter values obtained from fits of the 2C3K and 3C5K models to single voxel TAC data simulated by adding voxel‐level noise to ground‐truth 2C3K or 3C5K models. Values of *MB*, σ_*B*_, σ_*P*_ *,* and σ_*T*_ are shown for fitted parameters as percentages of the mean values of directly related ground‐truth parameters of the 2C3K or 3C5K models. When no directly related parameter exists, σ_*P*_ is shown alone as a percentage of the mean fitted parameter value

Ground‐truth 2C3K model							
Model fitted		Fitted model parameters					
2C3K	*v*_B_	*K*_1_	*k*_2_			*k*_3_	*k*_*flux*_
*MB* (%)	−12	−4	−4			−4	−4
σ_*B*_ (%)	13	7	7			6	5
σ_*P*_ (%)	36	20	28			37	29
σ_*T*_ (%)	39	21	29			37	30
3C5K	*v*_B−3C_	*K*_1−3C_	*k*_2−3C_	*k*_3−3C_	*k*_4−3C_	*k*_5−3C_	*k*_*flux*−3C_
*MB* (%)	−22	–	–	–	–	398	−5
σ_*B*_ (%)	39	–	–	–	–	591	11
σ_*P*_ (%)	43	199	155	61	64	89	32
σ_*T*_ (%)	58	–	–	–	–	597	34

Ground‐truth 3C5K model							
Model fitted		Fitted model parameters					
2C3K	*v*_B_	*K*_1_	*k*_2_			*k*_3_	*k*_*flux*_
*MB* (%)	53	–	–			−42	24
σ_*B*_ (%)	85	–	–			56	37
σ_*P*_ (%)	44	32	42			33	31
σ_*T*_ (%)	96	–	–			65	47
3C5K	*v*_B−3C_	*K*_1−3C_	*k*_2−3C_	*k*_3−3C_	*k*_4−3C_	*k*_5−3C_	*k*_*flux*−3C_
*MB* (%)	−5	25	104	90	67	27	−1
σ_*B*_ (%)	15	174	535	208	104	58	13
σ_*P*_ (%)	56	188	227	119	125	142	44
σ_*T*_ (%)	58	257	581	240	162	153	47

Fits of the 2C3K model to two example TACs are plotted in Fig. 3. An example of a voxel TAC fitted better by 3C5K than 2C3K is shown in Fig. S1. An example fit of Feng's input function to an image‐derived blood TAC is shown in Fig. S2. Mean fitted model parameter values for all 24,662 voxels are shown in Table 5.

**Table 5:** Mean and standard deviations of fitted 2C3K model parameter values for all voxel TACs

2C3K	*v*_B_ (%)	*K*_1_ (mL min^−1^g^−1^)	*k*_2_ (min^−1^)	*k*_3_ (min^−1^)	*k*_*flux*_ (mL min^−1^g^−1^)
Mean	4.1	0.20	0.25	0.0028	0.0019
SD	11.4	0.19	0.18	0.0027	0.0022

### 3.B. Clustering of fitted rate‐constants

**Figure 4:**
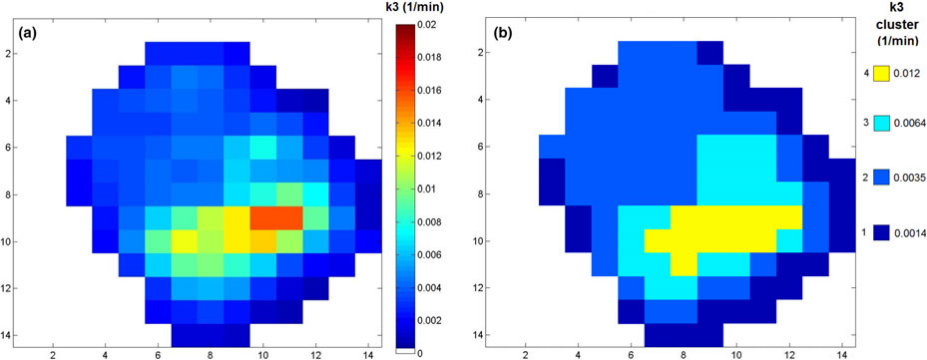
A slice taken from the *k*_*3*_ parametric map obtained for Patient 6 pre‐Buparlisib, showing (a) unclustered and (b) clustered *k*_3_ values.

Values obtained from fits to the 24,662 voxels clustered into 6 independent levels for the 2C3K model parameter *v*_B_, 14 independent levels for *K*_1_, 7 independent levels for *k*_2_, and 4 independent levels for *k*_3_. Figure 4 shows a slice through the tumor parametric map obtained for *k*_3_ (the nominal rate‐constant for FMISO binding) for Patient 6 imaged pre‐Buparlisib, together with the clustered version of the map. Although unclustered maps contain a broad and continuous range of parameter data, the clustered maps provide a representation in which the data is grouped into a discrete set of levels, chosen to best represent the voxel data taking into consideration the individual fitted parameter values and known total uncertainties on them. A set of parameter values having large uncertainties and covering a certain range will be grouped into fewer clusters by our scheme than another set covering the same range but with smaller uncertainties, reflecting the reduced information of the more uncertain dataset.

**Figure 5:**
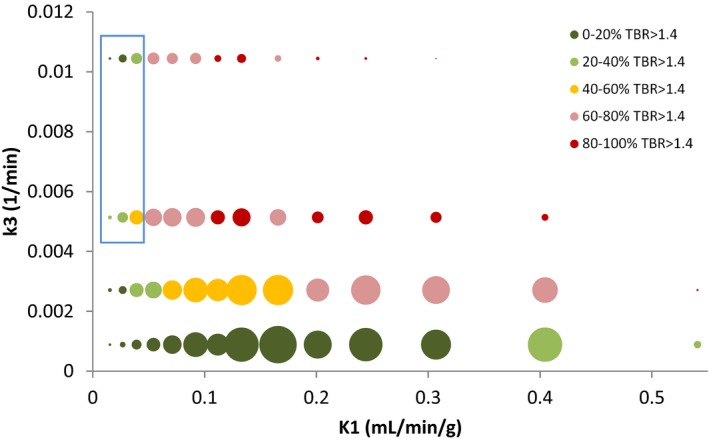
Bubble plot showing clustered *k*_3_ versus *K*_1_ values. Bubble size indicates the number of points in each cluster. Bubbles are colored according to the proportion of voxels with *TBR* (4 h p.i.) > 1.4. The blue box highlights a region of the graph in which *k*_3_ values are high, but less than 60% (generally < 40%) of voxels have *TBR *> 1.4.

A bubble plot of numbers of voxels assigned to *K*_1_ and *k*_3_ cluster levels is shown in Fig. 5. The size of each bubble represents the number of voxels contributing to that data‐point. The bubble color represents the proportion of voxels contributing to the bubble that have *TBR*s > 1.4 at 4 h p.i., the general consensus when interpreting FMISO PET images being that voxels with *TBR*s > 1.4 are hypoxic.^30^ From the figure, it can be seen that bubbles containing higher percentages of voxels with *TBR*s > 1.4 generally also have larger mean *k*_3_ values, as expected since the rate‐constant for FMISO binding is greater in hypoxic tissues.^1^, ^2^, ^3^ However, bubbles within the blue box region of Fig. 5 have low fractions of voxels with *TBR*s > 1.4, but high mean *k*_3_ and very low mean *K*_1_ values which might be considered to represent hypoxia caused by very poor perfusion. Presumably in this region, the availability of tracer for intracellular reduction was limited by the very low perfusion levels, thereby reducing *TBR*s.

### 3.C. Variation in weighted mean rate‐constants with distance from the tumor edge

**Figure 6:**
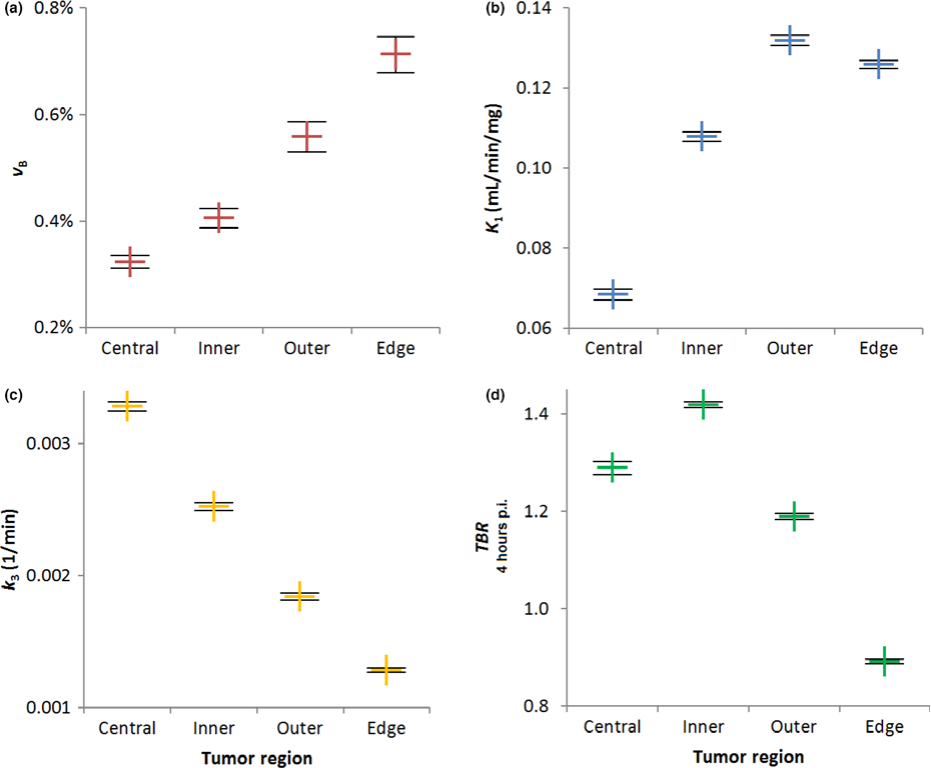
Variation with distance from the tumor edge of weighted mean values of (a) *v*_B_, (b) *K*_1_, (c) *k*_3_, and (d) *TBR* parameters for all patients pre‐Buparlisib. Error bars show standard errors on the mean. [Color figure can be viewed at wileyonlinelibrary.com]

Weighted means of fitted *v*_B_, *K*_1_, and *k*_3_ parameter values are plotted against distance from the tumor edge for all voxels imaged pre‐Buparlisib in Fig. 6, together with weighted mean *TBR* values at 4 h p.i. Example images of clustered *v*_B_, *K*_1_, *k*_3_, and *TBR* values are shown for two patients in Fig. S3.

Moving inwards from the edge, weighted mean values of *v*_B_, the fractional blood volume, fall significantly (*P* < 0.001) between successive voxel layers. *K*_1_ values also fall significantly (*P* < 0.0001) between the *outer* and *inner* layers, and between the *inner* and *central* layers, as might be expected. However, between the *edge* and *outer* voxel layers, *K*_1_ rises significantly (*P* < 0.001) despite *v*_B_ falling.

Again moving inwards from the tumor edge, weighted mean *k*_3_ values rise significantly (*P* < 0.0001) between each successive layer of voxels. Weighted mean *TBR* values also rise significantly (*P* < 0.0001) between the *edge* and *outer* layers, and between the *outer* and *inner* voxels, but then fall significantly between the *inner* and *central* layers (*P* < 0.0001).

**Figure 7:**
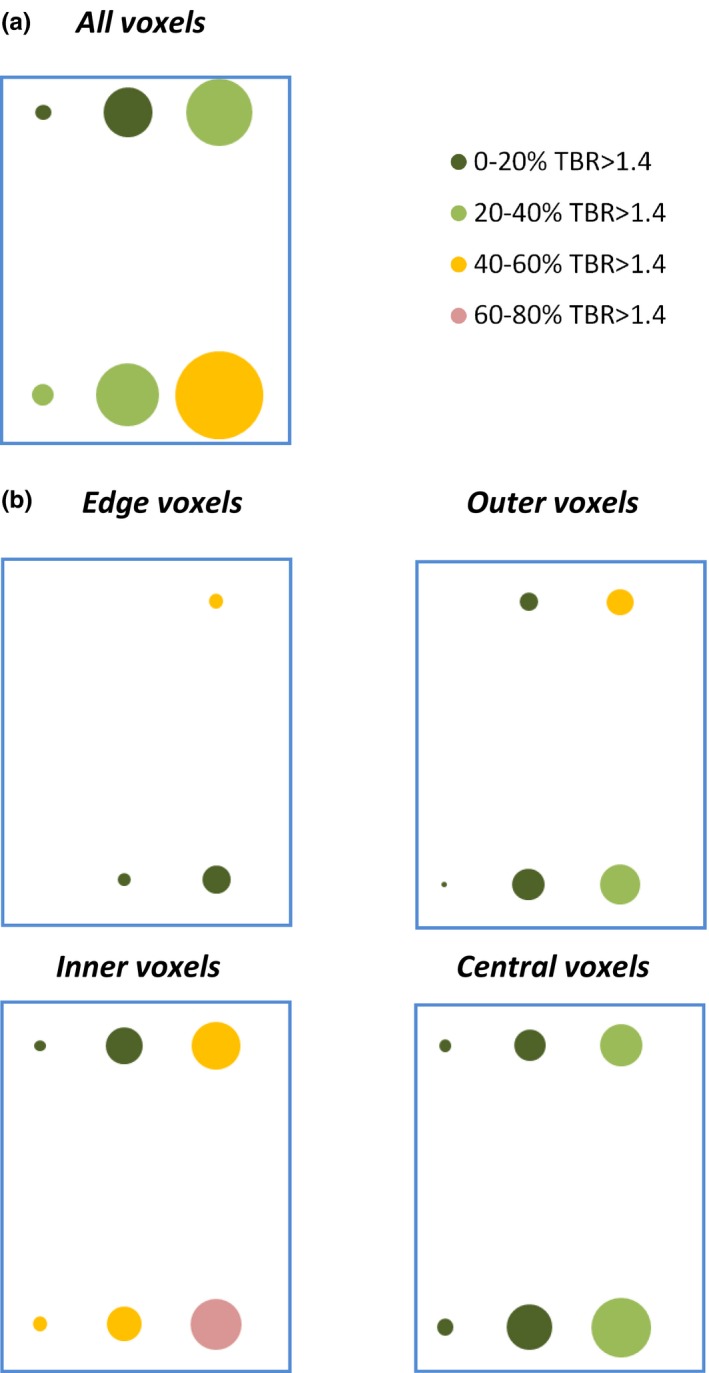
(a) Zoom of the blue box region of Fig. 5, showing clustered *k*_3_ versus *K*_1_ values for all voxels. (b) The same data, now split into tumor regions. Bubbles are colored according to the proportion of voxels with *TBR* > 1.4 (4 h p.i.). [Color figure can be viewed at wileyonlinelibrary.com]

Figure 7 shows the blue box region from Fig. 5 split into distance categories (the full version of Fig. 5 split into distance categories is shown in Fig. S4). Most voxels within this region are drawn from the *inner* and *central* tumor layers. In the *central* layer, voxel kinetics are particularly mismatched with uptake levels, only 23% of these voxels having *TBR*s > 1.4 despite their high *k*_3_ and very low *K*_1_ values. This mismatch mirrors the decrease in *TBR* values seen in Fig. 6 between voxels in the *inner* and *central* tumor regions, despite the increase in mean *k*_3_ and decrease in mean *K*_1_ values seen between the two layers.

## 4 Discussion

An irreversible two‐tissue compartment model, 2C3K, was found to be most useful for fitting FMISO uptake TACs of individual voxels of NSCLC tumors. Although information criterion scores were slightly worse for 2C3K than for 3C5K fits to single voxel TACs, in statistical simulations 2C3K model fits provided more precise parameter estimates overall than 3C5K fits. The 2C3K model and variants have been used in previous analyses of FMISO uptake kinetics, and we will use the model in voxel‐by‐voxel kinetics analyses of tumor response to Buparlisib, generating data on the spatial variation in the response.

It is possible to estimate the expected rate‐constants from FMISO within human tissues. Capillary surface areas 50–260 cm^2^ g^−1^ have been reported.^31^ The surface area‐to‐volume ratio for a cylindrical blood vessel, diameter d, is 4/d and so for a typical diameter 30 μm equals 1.3 × 10^3^ cm^−1^ 32 This implies a *v*_B_ = (50−260)/1.3 × 10^3^ cm^3^ g^−1^ = 4–15%,^31^ which is comparable to that used in our simulations (Table 3). The vessel permeability coefficient for sucrose, molecular weight (MW) 342 amu, is 1 × 10^−5^ cm s^−1^ 31 FMISO has a MW of 188 amu so this value could be used as an estimate of FMISO vessel wall permeability. The rate‐constant *K*_1−3C_ can be shown to approximately equal the vessel wall permeability multiplied by the vessel surface area‐to‐volume ratio.^32^ This would give 0.8 min^−1^ as an estimate of *K*_1−3C_, broadly similar to the value in our simulations. FMISO diffuses rapidly within cells so *k*_3−3C_ would be of the same order as *K*_1−3C._ 2 Rates of FMISO binding from clinical data have been estimated at 0.001 min^−1^ for normoxic tissue and 0.005 min^−1^ in hypoxia tissue,^17^ which is similar to the 0.002 min^−1^ mean *k*_3_ (and 0.003 min^−1^ mean *k*_5−3C_) used in our simulation. The 2C3K rate‐constant values in Table 3 are also broadly similar to those used in the simulations by Wang et al.^27^ This suggests that the parameters used for the statistical simulation are physiologically reasonable. These values are also very similar to the mean values for all analyzed voxels (Table 5).

When voxels were clustered by their fitted *K*_1_ and *k*_3_ values, a mismatch was observed between the clustered rate‐constants and proportions of voxels with *TBR*s > 1.4. The mismatch occurred in a region with high *k*_3_ and very low *K*_1_ rate‐constants, a combination readily interpretable as indicating hypoxia due to poor perfusion, and yet less than quarter of all voxels in the region had *TBR*s > 1.4. Presumably, the mismatch arose because the quantity of tracer available for uptake was fundamentally limited by the poor perfusion. If tumor hypoxia status was reported purely on the basis of *TBR* values, the mismatch would potentially lead to an underestimation of hypoxic volume.

We categorized voxels according to their distances from the tumor edge and calculated weighted mean parameter values for each distance category (Fig. 6). The weighted mean fractional vascular volume, *v*_B_, fell with distance from the tumor edge while *k*_3_ rose, findings that would be expected if more central tumor regions were less vascularized (lower *v*_B_) and more hypoxic (higher *k*_3_). No previous work appears to have been published demonstrating this distance dependence based on PET kinetic modeling, although previous work using perfusion CT has shown permeability and fractional blood volumes to be higher at tumor edges than at their centers.^33^ The lack of PET studies exploring this distance dependence is likely due to the fact that many of the dynamic hypoxia PET studies reported to date have been carried out for relatively small tumors, such as from head‐and‐neck cancer.

Weighted mean *TBR* values rose with distance from the tumor edge, except between the *inner* and *central* layers where *TBR* fell. Further analysis showed that many of the voxels with mismatched *TBR*s and rate‐constants were drawn from the *central* layer, indicating that the fall in *TBR* but rise in *k*_3_ observed in this layer was due to the same mismatch, and suggesting that the degree of hypoxia at tumor centers might be underestimated using the *TBR* measure alone (Fig. 7). A mismatch between *TBR*s and *K*_1_ and *k*_3_ values has been observed before,^15^ and proposed as a motivation for performing kinetics analysis, but the mismatch was not spatially localized within tumors.

The gold‐standard method for determining input functions is direct arterial line sampling. Here we used image‐derived input functions (IDIFs) calculated from mean tracer activity concentrations within volumes drawn in the descending aorta, both for patient comfort and safety, and because good agreement has been demonstrated between directly sampled input functions and IDIFs obtained from the descending aorta.^20^ FMISO is metabolically stable in blood and so metabolite correction is not required,^18^ and plasma over blood ratios are equal (1) and constant.^34^ As with any IDIF there is a possibility for bias from partial volume and spill in effects. However, to minimize these effects the volumes‐of‐interest used to obtain IDIFs were located centrally within the descending aorta.

Errors in voxel TACs may be introduced by the registration process between the images collected during the first 45 min and a 2 and 4 h scans p.i. Clearly, it is not feasible to keep a patient on the scanner couch for 4 h, and so images were collected in three separate sessions during each complete dynamic FMISO study. Any errors were minimized by getting the patient set up in the same position for each part of the study, generally by the same member of staff, and also by performing rigid CT‐to‐CT registration followed by manual tumor‐to‐tumor matching.

As the tumors investigated were situated in the thorax it is possible that respiratory movement during the PET acquisition could also impact the voxel TACs. Patients were 4DCT imaged alongside the PET imaging, and the maximum tumor movement observed in these 4DCT scans was 5 mm in the cranial‐caudal (CC) direction for a tumor length of 60 mm in the same direction. The average CC movement for tumors of CC length 30–110 mm (median 75 mm) was 3 mm. Generally the tumors investigated were situated in the upper lobe, and such tumors have been shown to move less than lower lobe tumors.^35^

The degrees of vascularity and hypoxia will vary somewhat within each tumor voxel, due both to movement and because the length‐scales on which these quantities vary is smaller than the 5 mm voxel‐size.^36^ The kinetic models include a term (*v*_B_) to take into account the average vascular component within the voxel. The resolution of our PET system is approximately the same as the voxel size (5 mm), and so PET imaging is unable to resolve details smaller than this. Consequently, the results of the PET kinetic modeling describe the kinetics of FMISO uptake averaged over the voxel volume.

In this work, we have assessed the performance of kinetic models using a data‐led approach based on information criteria and statistical simulations. The kinetic models investigated here comprised linear chains of compartments. Branching compartment models may describe the processes involved in FMISO binding and reduction more accurately, however transformation analysis can sometimes be used to reduce branching models to mathematically equivalent linear representations. For example, a simplified version of the Casciari et al. model can be shown to be equivalent to the 3C5K model.

Recently, we have begun a new histological study in which surgically treated NSCLC patients are imaged using dynamic FMISO PET ahead of tumor excision. This will enable *k*_3_ and *TBR* from the FMISO PET to be correlated with pimonidazole staining to test the findings of this paper.

## 5 Conclusions

The kinetics of FMISO uptake in voxels of tumors in NSCLC patients were described more precisely by an irreversible two‐tissue compartment model, 2C3K, than more complex models. Simulation studies indicated a precision of 39% (1 SD) for fitted values of the tumor fractional blood volume (*v*_B_) and 37% for the FMISO binding rate‐constant (*k*_3_).

Weighted mean values of *v*_B_ fell significantly with distance from the tumor edge, while weighted mean values of *k*_3_ rose significantly. Moving toward tumor centers, *k*_3_ values continued to rise and *v*_B_ and *K*_1_ to fall, all conceptually indicative of increasing hypoxia; however, *TBR* indices at 4 h p.i. fell toward tumor centers. Thus, assessments of FMISO images made on the basis of FMISO *TBR* alone (a classic imaging marker of hypoxia) may underestimate the extent of hypoxia, particularly at tumor centers.

## Conflicts of interest

The authors declare they have no conflicts of interest.

## Supporting information


**Figure S1**. Example voxel TAC fitted better by the 3C5K model than 2C3K. Time post‐injection is plotted on linear and logarithmic scales.

**Figure S2**. Fit of the Feng input function to an example blood TAC. Time post‐injection is plotted on logarithmic and linear scales.

**Figure S3**. Example axial slices through two tumors showing clustered values for *v*_B_, *K*_1_, *k*_3_, and *TBR*. The colors in the plots indicate which cluster levels each voxel has been assigned to, with cluster level 1 being the lowest value for each parameter.

**Figure S4**. Bubble plots showing clustered *k*_3_ versus *K*_1_ values. Bubble size indicates the number of points in each cluster. Bubbles are colored according to the proportion of voxels with *TBR* (4 h p.i.) > 1.4. Each plot is separated out to include voxels from each distance category.
