# Supplementary material for: 18F‐fluoromisonidazole uptake in advanced stage non‐small cell lung cancer: A voxel‐by‐voxel PET kinetics study
Source: Med Phys. 2017 Jul 21;44(9):4665–76. doi: 10.1002/mp.12416 (PMC5600259; doi:10.1002/mp.12416)

***Supplementary Figure 1*** – Example voxel TAC fitted better by the 3C5K model than 2C3K. Time post-injection is plotted on linear and logarithmic scales.

**
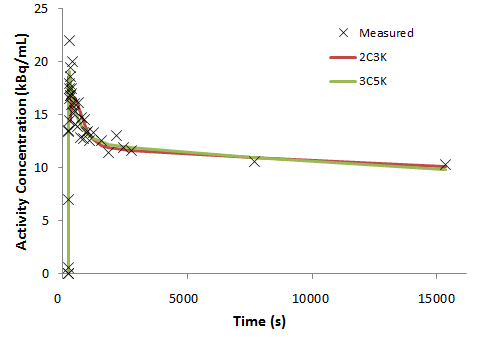
**


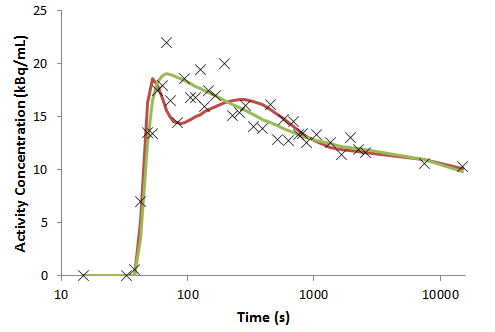

Supplement: Supplementary file 1 — Figure S1. Example voxel TAC fitted better by the 3C5K model than 2C3K. Time post‐injection is plotted on linear and logarithmic scales. [file MP-44-4665-s001.doc]
