# Supplementary material for: 18F‐fluoromisonidazole uptake in advanced stage non‐small cell lung cancer: A voxel‐by‐voxel PET kinetics study
Source: Med Phys. 2017 Jul 21;44(9):4665–76. doi: 10.1002/mp.12416 (PMC5600259; doi:10.1002/mp.12416)

***Supplementary Figure 2*** – Fit of the Feng input function to an example blood TAC. Time post-injection is plotted on logarithmic and linear scales.


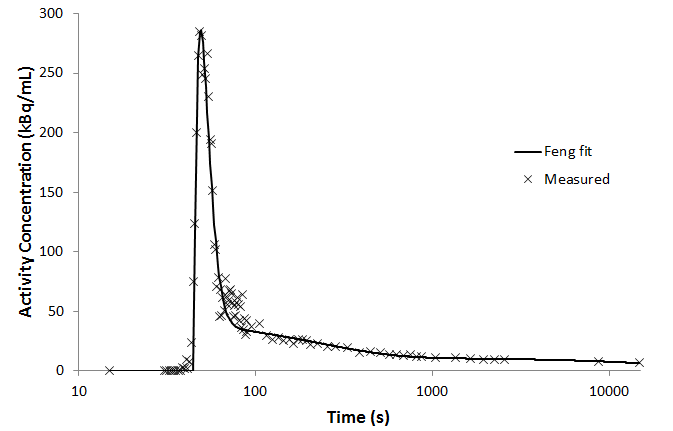


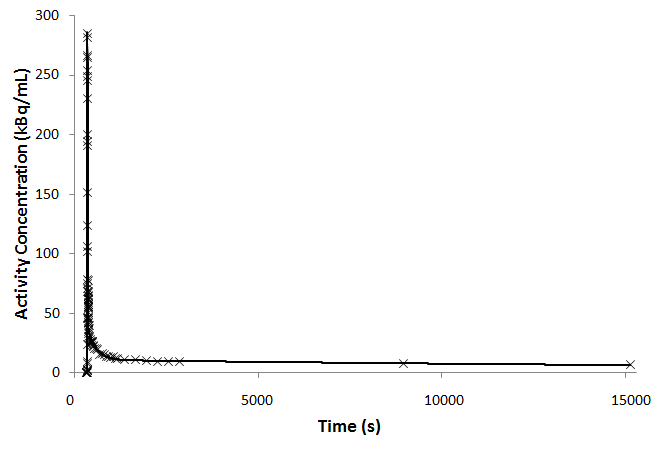

Supplement: Supplementary file 2 — Figure S2. Fit of the Feng input function to an example blood TAC. Time post‐injection is plotted on logarithmic and linear scales. [file MP-44-4665-s002.doc]
