# Supplementary material for: 18F‐fluoromisonidazole uptake in advanced stage non‐small cell lung cancer: A voxel‐by‐voxel PET kinetics study
Source: Med Phys. 2017 Jul 21;44(9):4665–76. doi: 10.1002/mp.12416 (PMC5600259; doi:10.1002/mp.12416)

***Supplementary Figure 3*** – Example axial slices through two tumors showing clustered values for *v*_B_, *K*_1_, *k*_3_, and *TBR*. The colors in the plots indicate which cluster levels each voxel has been assigned to, with cluster level 1 being the lowest value for each parameter.

**Patient 3 Pre-Buparlisib**


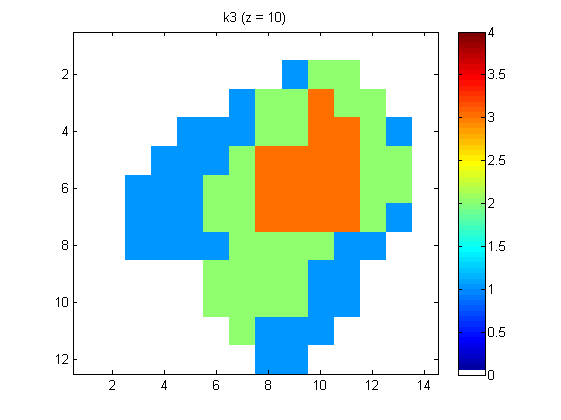

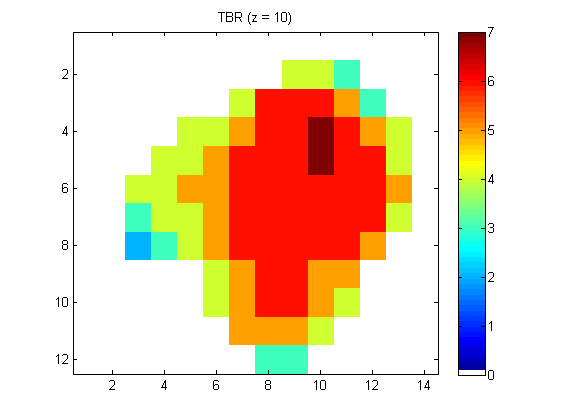


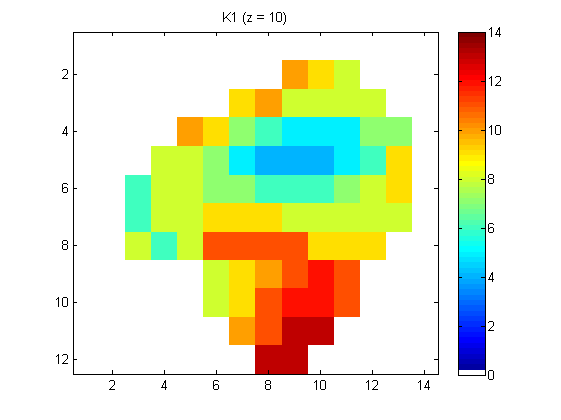

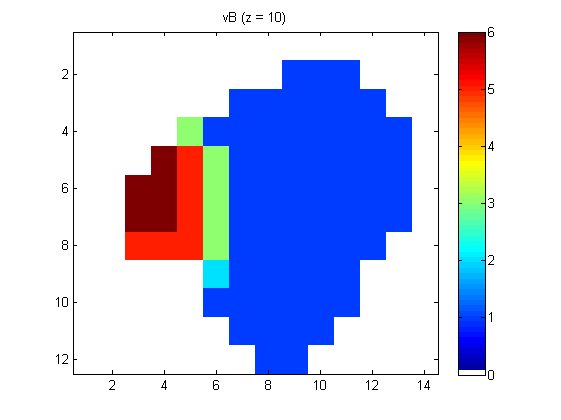


**Patient 5 Pre-Buparlisib**


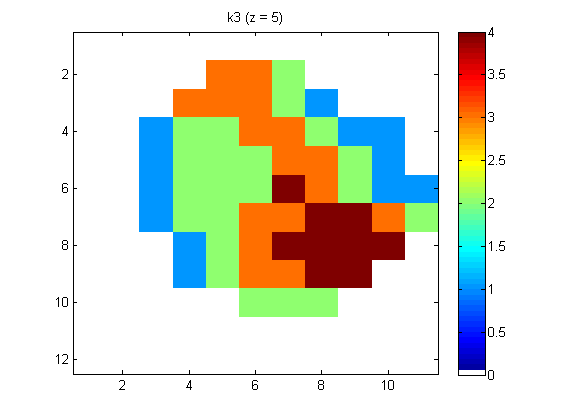

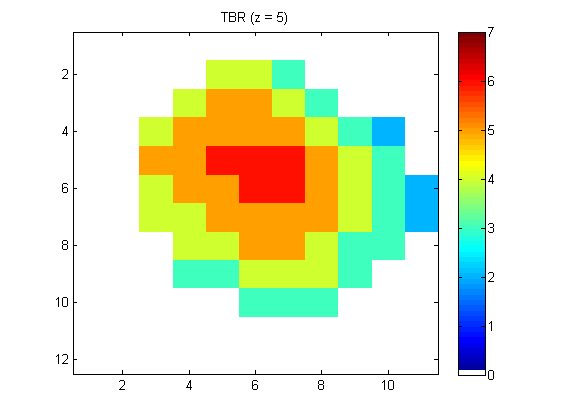


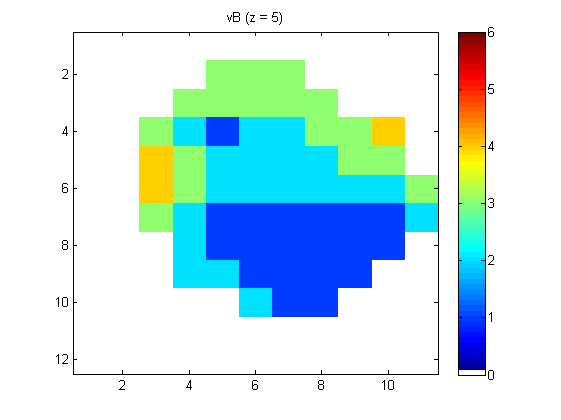

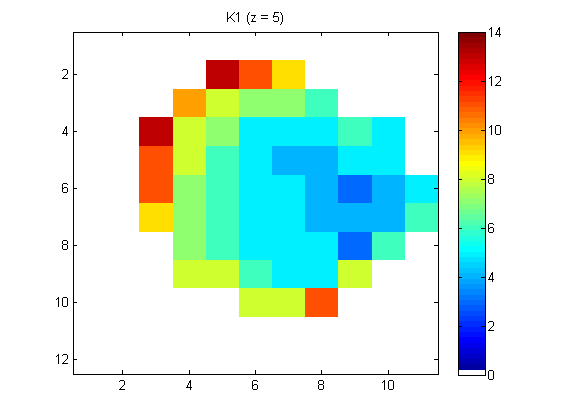

Supplement: Supplementary file 3 — Figure S3. Example axial slices through two tumors showing clustered values for v B, K 1, k 3, and TBR. The colors in the plots indicate which cluster levels each voxel has been assigned to, with cluster level 1 being the lowest value for each parameter. [file MP-44-4665-s003.doc]
