# Supplementary material for: 18F‐fluoromisonidazole uptake in advanced stage non‐small cell lung cancer: A voxel‐by‐voxel PET kinetics study
Source: Med Phys. 2017 Jul 21;44(9):4665–76. doi: 10.1002/mp.12416 (PMC5600259; doi:10.1002/mp.12416)

***Supplementary Figure 4*** – Bubble plots showing clustered *k*_3_ versus *K*_1_ values. Bubble size indicates the number of points in each cluster. Bubbles are colored according to the proportion of voxels with *TBR* (4 hours p.i.) >1.4. Each plot is separated out to include voxels from each distance category.

**Edge voxels**


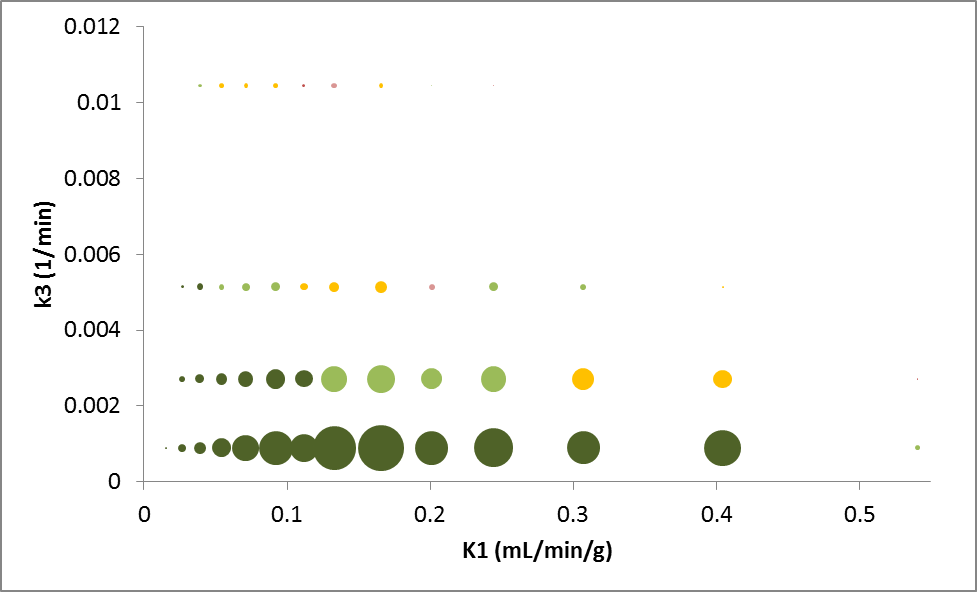


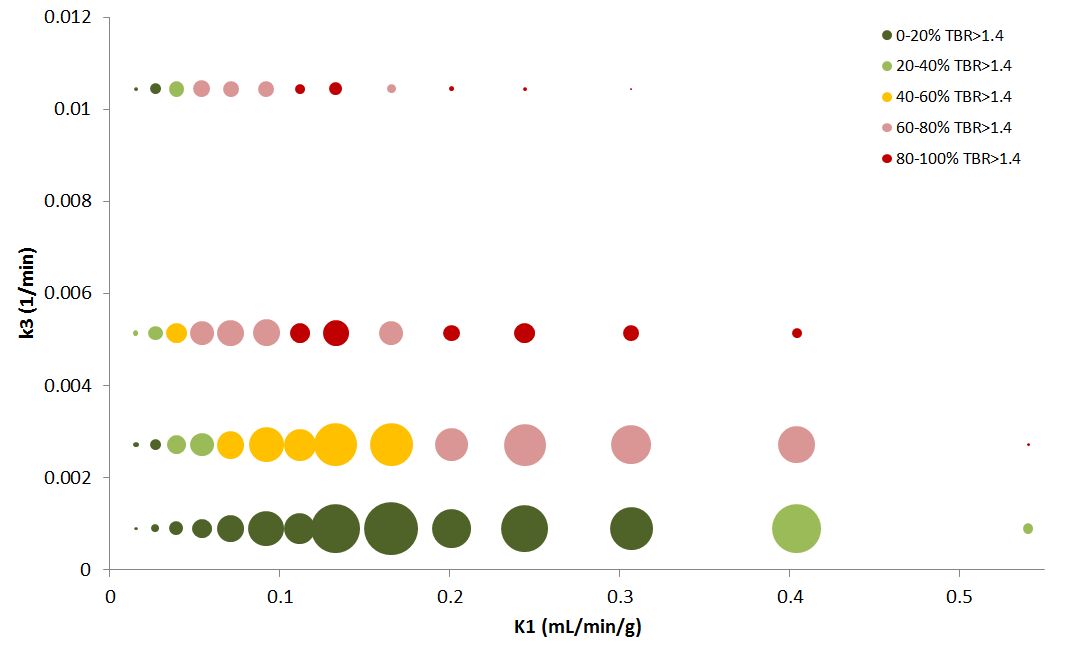


**Outer voxels**


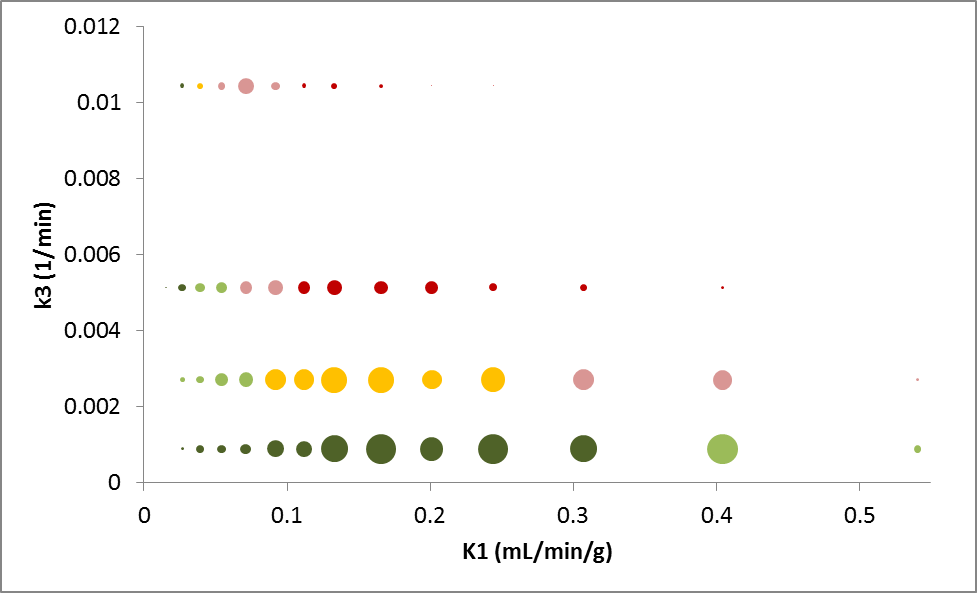


**Inner voxels**


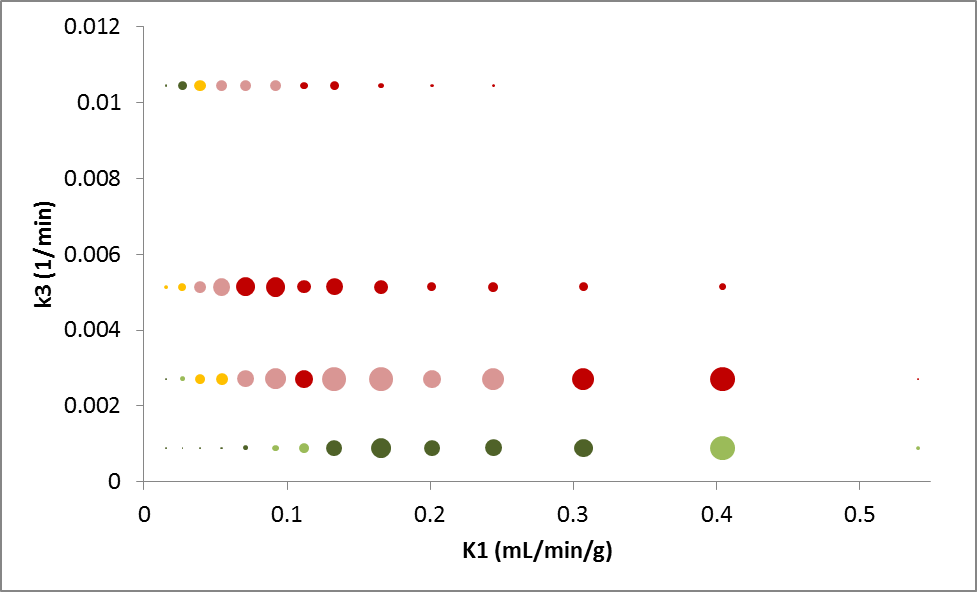


**Central voxels**


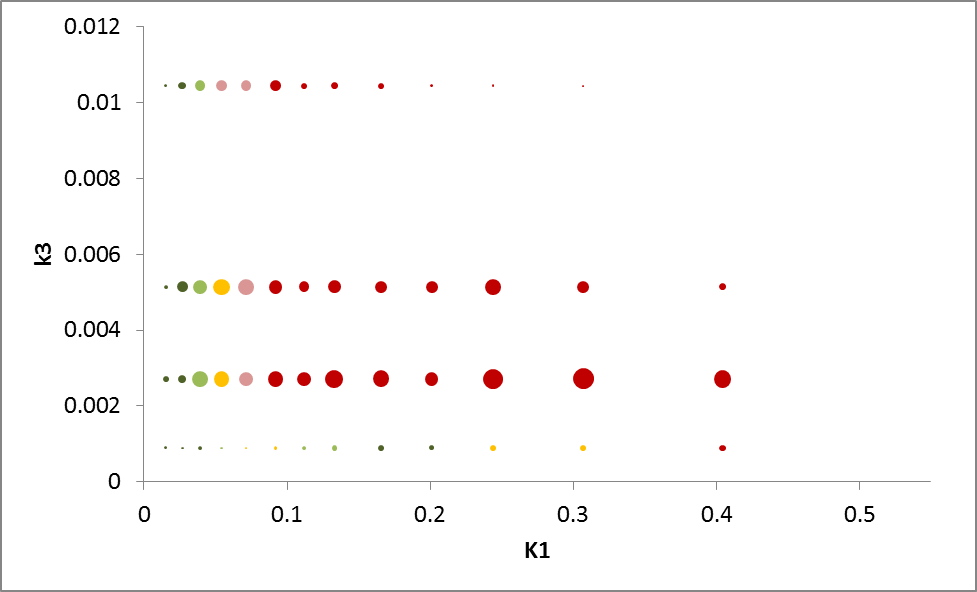

Supplement: Supplementary file 4 — Figure S4. Bubble plots showing clustered k 3 versus K 1 values. Bubble size indicates the number of points in each cluster. Bubbles are colored according to the proportion of voxels with TBR (4 h p.i.) > 1.4. Each plot is separated out to include voxels from each distance category. [file MP-44-4665-s004.doc]
